# Modes of Early Detection of Breast Cancer in Katowice Region, Poland

**DOI:** 10.3390/ijerph17082642

**Published:** 2020-04-12

**Authors:** Jan Eugeniusz Zejda, Angelina Kaleta

**Affiliations:** Department of Epidemiology, School of Medical Sciences in Katowice, Medical University of Silesia, 40-752 Katowice, Poland; jzejda@sum.edu.pl

**Keywords:** breast cancer, early diagnosis, survivors

## Abstract

Background: Our 2004 survey of breast cancer survivors in the Katowice region (Poland) showed that the detection of the disease was triggered by self-examination in 58.9%, mammography in 19.2%, and clinical examination in 19.7% of cases. The purpose of the current study (2019/2020) was to determine if the implementation of national screening (mammography) in 2007 resulted in an increase of the relative contribution of mammography to detection of cancer. Methods: Subjects were 215 breast cancer patients, members of self-support groups in Katowice region. The questionnaire included questions on early detection of breast cancer, participation in screening, and socio-economic status. Results: Early detection of cancer was initiated by self-examination in 63.7%, mammography in 22.8%, clinical examination in 13.5% of subjects. Age at detection depended on the method (*p* < 0.001): 54.1 ± 10.8 years for self-examination, 60.0 ± 7.6 years for mammography, and 58.7 ± 10.8 years for clinical examination. Conclusions: Both in 2004 and 2020 self-examination is the most frequent method of early detection of breast cancer in the study area. The contribution of mammography remains on a low level (23%). This finding could be explained by a low participation in screening and by age of 50 years used as the entry criterion to national screening of breast cancer in Poland.

## 1. Introduction

In Poland, breast cancer is the second leading cause of cancer death in women with annual (2017) age-adjusted incidence rate equaling 48.6/100,000 and mortality rate equaling 16.7/100,000 [1]. The burden of the disease has not diminished over the recent decades. On the contrary, in 2000 the incidence was 38.8/100,000 and mortality was 15.0/100,000 [1]. An important factor contributing to epidemiological profile of the disease is the survival rate. In Poland its current value of 76.5% in five years is approximately 6% points lower than the average rate in European Union [1,2]. 

The chances for successful treatment of breast cancer depend on the clinical stage of the tumor. Consequently, early diagnosis improves the treatment outcomes and is critical to survival. Some factors that are pertinent to early diagnosis include awareness, access to screening and care, and health system capacity and resources. In general the tools of early diagnosis of breast cancer include breast self-examination, clinical breast examination and mammography that in a screening setting was shown to reduce breast cancer mortality by 20% [3]. It is unclear to what extent the epidemiological picture of breast cancer in Poland depends on the actual mode of the detection of the disease. Our 2004 survey including 236 breast cancer survivors in Katowice region showed that diagnosis of the disease was triggered by self-examination in 58.9%, mammography in 19.2% and clinical examination in 19.7% of cases [4]. The survey was performed in years when the population-based screening for breast cancer was not available, in the region. National screening for breast cancer was introduced in 2007 and is offered free of charge to women aged 50–69 years [5]. This preventive measure and its country-wide promotion as well as increasing access to mammography outside of the national screening programs might have affected the modes of early detection of breast cancer. With this caveat in mind we decided to repeat the survey in Katowice region to describe the current modes of detection of the disease and to compare the current findings with those analogous, obtained 15 years ago. The purpose of our study was to find out the modes of early detection of breast cancer and to determine if the implementation of national screening (mammography) in 2007 resulted in an increase of the relative contribution of mammography to diagnosis of the disease.

## 2. Materials and Methods

The study subjects were women registered with self-support groups of patients after mastectomy due to breast cancer (“Amazonki”). Eight “Amazonki groups” active in the Katowice region were contacted and invited to participate in the survey. In the period between September 2019 and January 2020 a total of 478 women received anonymous questionnaires. The questions dealt with socio-economic status, age, and method of the detection of breast cancer, participation in population-based mammography, family history of breast cancer. The completed questionnaires were returned by 215 women thus resulting in a 45% participation rate.

Statistical analysis of data involved distribution of quantitative and qualitative variables. Nonparametric Kruskal–Wallis test was used to assess statistical significance of differences between distributions of quantitative variables and a chi-square test was applied to assess the differences in distributions of qualitative variables. The value *p* < 0.05 was used in statistical inference. 

The study protocol was approved by University Ethics Committee (decision KNW/0022/KB1/114/19).

## 3. Results

The subjects were 215 women aged 32–88 years (mean: 66.0 ± 9.9 years). Almost all women (97.7%) lived in towns, majority (70.7%) had at least secondary-level education, 65% were married. Family history of breast cancer was reported by 41% of subjects, with a first degree of relationship declared by 29.8% of subjects.

**Figure 1:**
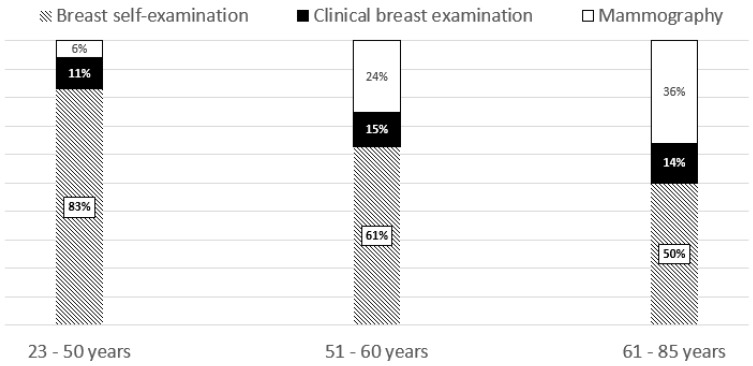
Modes of detection of breast cancer suspected changes according to age at diagnosis.

Average age at diagnosis was 56.1 ±10.5 years and varied between 23 and 85 years. The mean time since the diagnosis was 10.1 ± 7.1 with the range 0–37 years. Among three analyzed modes of early detection of breast cancer self-examination was the most frequent method (63.7%), followed by mammography (22.8%) and clinical breast examination (13.5%). The three groups differed (*p* < 0.001) in terms of age at diagnosis: 54.1 ± 10.8 years in breast self-examination group, 58.7 ± 10.8 years in clinical breast examination group and 60.0 ± 7.6 years in mammography group. Based on the age at diagnosis three age categories were defined: 23–50 years (n = 63; 29.3%), 51–60 years (n = 82; 38.1%) and 61–85 years (n = 70; 32.6%). Figure 1 shows the relative contributions of three examinations according to the age at diagnosis. With increasing age category there was a decreasing frequency of breast self-examination and increasing frequencies of two remaining modes of diagnosis. The differences were statistically significant: *p* = 0.001.

Table 1 shows the distribution of the modes of detection of breast cancer according to the education level, employment and marital status, and family history of breast cancer, at the age of diagnosis. Statistically significant effect was seen in relation to marital status. Breast self-examination was the most prevalent method of arriving at diagnosis of cancer among married women, whereas mammography played such a role mostly in single women and widows.

**Figure 2:**
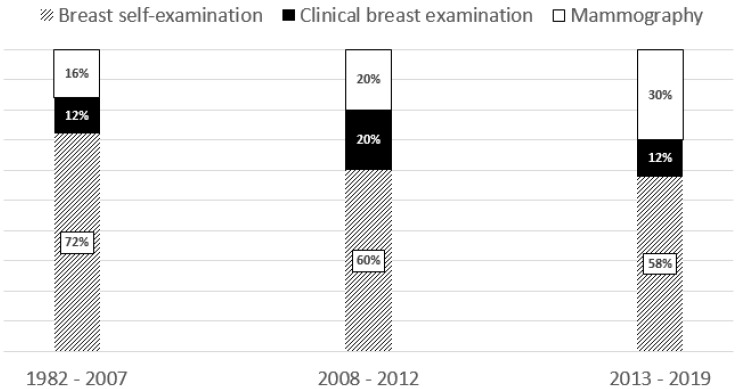
Modes of detection of breast cancer suspected changes according to year of diagnosis.

All diagnoses were made in the years 1982–2019. Figure 2 compares the distributions of diagnostic modes between three periods, years 1982–2007 (82 women), years 2008–2012 (41 women), and years 2013–2019 (92 women). The first cut-off (year 2007) was defined by the year of introduction of population-based screening for breast cancer and the second cut-off (year 2012) was defined by the fifth year of experience with population-based screening in Poland. The figure shows a gradually increasing contribution of mammography to diagnosis of the disease, from 16% in the first period to 30% in the last period but the difference was not statistically significant (*p* = 0.1).

In the study group 88 subjects had a family history of breast cancer and 127 subjects had no such history. As shown in Table 2 both groups were similar in terms of breast clinical examination as the cancer detection method. Mammography was more frequent in subjects with family history of breast cancer whereas self-examination was more frequent in subjects without family history of breast cancer, but the difference between the distributions of modes of detection was not statistically significant (*p* = 0.1).

In the examined group 133 women (61.9%) were diagnosed for breast cancer after 2007, since when a population-based screening program was available free of charge for women aged 50+ years and widely promoted via media. In that subgroup 81 women met the age-defined criteria of target population and out of them 51 (62.3%) knew about the program, and 26 (32.1%) received an invitation for a mammography. All who were invited underwent the mammography offered within the screening program.

## 4. Discussion

The principal finding of our study is that in the study population of breast cancer survivors (Katowice Region, Poland) breast self-examination remains the most frequent method leading to the detection of breast cancer. Results obtained in 2019 showed that breast self-examination led to diagnosis in 63.7%, clinical examination in 13.5% and mammography in 22.8% of cancer cases. The findings provided by study performed in 2004 showed similar figures: breast self-examination in 58.9%, clinical examination in 19.7% and mammography in 19.2% of cases [4]. The compared distributions (year 2004 vs. year 2019) did not differ in a statistically significant way: *p* = 0.1. Moreover the group examined in 2019 and the group examined in 2004 were similar in terms of age at diagnosis: 56.1 ± 10.5 years and 57.6 ± 9.3 years, respectively. Our observation that breast self-examination has remained the most frequent method of detection of breast cancer corresponds with widely known recommendations regarding a need for regular self-examination. The method is convenient and useful but it cannot replace other ways of early detection of breast cancer, in particular mammography [6]. From that point of view it is somewhat alarming that in the study group mammography was responsible for only 23% of all detected cases. Moreover, positive mammography results were obtained in cancer patients who were on average six years older than the cases identified by self-examination. The gap might reflect a reduced chance of successful management particularly that not a self-examination but a serial screening with mammography is the most effective method to detect early stage disease [7,8,9]. On the other hand such a low ratio of mammography-delivered early diagnoses in our study could reflect the age criterion used in population-based screening program. It is mammography and not self-examination that is effective in reducing breast cancer mortality [10]. Accumulated evidence was essential in revision of American Cancer Society guidelines that since 2015 advise beginning of annual mammography screening in women aged 40–44 years and recommend annual mammography screening in all women aged 45–54 years, followed by regular screening in women aged 55+ years [11]. Our findings showed that with regard to U.S. recommended age categories 16 women (7.4%) were aged 40–44 years, 69 women (32.1%) were aged 45–54 years and 120 women (55.8%) were aged 55+ years. The first two figures reveal the potential of increase in early detection in response to changes of the age scheme of population-based screening for breast cancer in Poland. 

In the examined group of 215 subjects 81 women had diagnosis made after 2007 and were aged 50 years or more at the time of diagnosis. In other words this subgroup of 81 women qualified for a population-based screening program. The fact is that only one third of them participated in the program. This finding identifies an important problem related to the organization and execution of population-based screening of breast cancer in Poland. Some centers registered with the program issue invitations to women residing in their catchment area and in our study 26 out of above mentioned 81 women received and accepted an invitation. The rule however is that the participation depends on individual initiatives and is encouraged by media-related campaign. A recent independent evaluation of the program identifies low accessibility to mammography centers in some regions of Poland and a very low participation rate ranging between 41% and 44% [12]. The latter conclusion is pertinent to our study’s population and justifies further public health efforts aiming at the improvement of screening outcomes. 

There are obvious limitations of our study’s findings on potential for generalization of the results. First of all we examined breast cancer survivors and only those who are active members of self-support “Amazonki groups”. As a result the distribution of modes of early detection of breast cancer remains unknown for other breast cancer patients, including those who are no longer alive. Secondly, our study had a relatively low participation rate, thus impairing a generalization of the results over all breast cancer survivors. Moreover the protocol of our study did not permit to include information about the type and stage of breast cancer. Hence it remains unknown if these important characteristics played any role in the group distribution of three modes of early detection of breast cancer. 

The rationale behind our survey was that the implementation of free-of-charge national screening would increase the contribution of mammography to early detection of breast cancer. Before the implementation of the population-based screening the mammography accounted for 19% of disease detection. Its current contribution to detection of breast cancer remains on a similarly low level of 23%. This finding might reflect the shortcomings of the screening program in Poland, resulting in a potentially serious consequences because it is mammography screening that may increase survival and reduce the risk of dying from breast cancer, even by about 40% [13,14]. The survival rate in Europe varies between the countries. For breast cancer diagnosed during 2010–2014 a five-year survival was 85% or above in 16 Western European countries and within the range 70–79% in Eastern European countries. In Poland, a five-year survival increased from 71.3% for cancer diagnosed in 2000–2004 to 76.5% for cancer diagnosed in 2010–2014. For comparison, over the same period in Germany five-year survival increased from 83.9% to 86.0%, and similar changes were reported for other Western European countries [2]. With regard to probable causes of apparently insufficient role of the population-based screening in Poland two factors should be taken into account: a low participation and a fixed age of 50 years as the entry criterion. Both factors are likely to explain an apparent underuse of the screening program of breast cancer in Poland and justify a revision of public health policy and practice in that field.

## 5. Conclusions

In conclusion, in breast cancer survivors in the Katowice region the most frequent mode of early detection of the disease is breast self-examination, followed by mammography and clinical examination. The relative contribution of three analyzed methods of breast cancer detection has not changed in a significant way between 2004 and 2020. The contribution of mammography remains on a low level of 23%, even though this figure was obtained 13 years after the implementation of national screening program. This finding can be attributed to a low participation in a population-based breast cancer screening program on the one hand and to an age of 50 years used as the entry criterion to that program, on the other hand. Both explanations deserve attention and action, and justify public health effort to revise the current protocol and performance of the population-based screening for breast cancer in Poland.

## Figures and Tables

**Table 1:** Modes of detection of breast cancer suspected changes according to the education level, employment and marital status, and family history of breast cancer, at the age of diagnosis.

Variable		Breast Self-Examination	Breast Clinical Examination	Mammography	*p*-Value *
Education Level	Primary School	39 (62.0%)	12 (19.0%)	12 (19.0)	0.3
	Secondary School	75 (65.2%)	15 (13.1%)	25 (21.7%)	
	University	23 (62.2%)	2 (5.4%)	12 (32.4%)	
Marital Status	Single	11 (42.3%)	6 (23.1%)	9 (34.6%)	0.03
	Married	100 (70.9%)	14 (9.9%)	27 (19.2%)	
	Widow	26 (54.2%)	9 (18.7%)	13 (27.1%)	
Employment	Employed	59 (72.0%)	9 (11.0%)	14 (17.0%)	0.2
	Unemployed	8 (53.3%)	4 (26.7%)	3 (20.0%)	
	Retired	70 (59.3%)	16 (13.6%)	32 (27.1%)	
Family History of Breast Cancer	Yes—I Degree	38 (59.4%)	8 (12.5%)	18 (28.1%)	0.1
	Yes—II Degree	11 (45.8%)	4 (16.7%)	9 (37.5%)	
	No	72 (68.6%)	13 (12.4%)	20 (19.0%)	
	Do not know	16 (72.7%)	4 (18.2%)	2 (9.1%)	

* Nonparametric Kruskal–Wallis test.

**Table 2:** Modes of detection of breast cancer suspected changes according to family history of breast cancer.

Variable		Breast Self-Examination	Breast Clinical Examination	Mammography
Family History of Breast Cancer	Yes	49 (55.7%)	12 (13.6%)	27 (30.7%)
	No	72 (68.6%)	13 (12.4%)	20 (19.0%)
	Do not know	16 (72.7%)	4 (18.2%)	2 (9.1%)
